# Predicting Individual Treatment Effects: Challenges and Opportunities for Machine Learning and Artificial Intelligence

**DOI:** 10.1007/s13218-023-00827-4

**Published:** 2024-01-22

**Authors:** Thomas Jaki, Chi Chang, Alena Kuhlemeier, The Pooled Resource Open-Access ALS Clinical Trials Consortium, M. Lee Van Horn

**Affiliations:** 1 University of Regensburg, Bajuwarenstraße 4, 93055 Regenburg, Germany; 2 University of Cambridge, Cambridge, UK; 3 Michigan State University, East Lansing, USA; 4 University of New Mexico, Albuquerque, USA

**Keywords:** BART, Heterogeneity in treatment effects, Personalized medicine, Predicted individual treatment effects

## Abstract

Personalized medicine seeks to identify the right treatment for the right patient at the right time. Predicting the treatment effect for an individual patient has the potential to transform treatment of patients and drastically improve patients outcomes. In this work, we illustrate the potential for ML and AI methods to yield useful predictions of individual treatment effects. Using the predicted individual treatment effects (PITE) framework which uses baseline covariates (features) to predict whether a treatment is expected to yield benefit for a given patient compared to an alternative intervention we provide an illustration of the potential of such approaches and provide a detailed discussion of opportunities for further research and open challenges when seeking to predict individual treatment effects.

## Introduction

Traditionally a clinical trial compares two treatments (experimental and control) based on an outcome (response) variable, *Y*. Data are gathered on an individual participant level, while decisions about treatment effectiveness are usually based on summaries (such as averages) of the individual data. Patients are, however, heterogeneous so that a patient’s individual characteristics (e.g., gender, disease severity, genetics) can lead to a patient’s personal treatment effect being markedly different from the average effect observed in trials. Analysis of (prespecified) subgroups can be used in the hope that an individual patient’s treatment effect is closer to the average within the subgroup. As such subgroups are usually defined on a very limited set of characteristics, however, such approaches still do not fully utilize all patient characteristics that modify the treatment effect. Subgroup analysis has also been criticized, as many approaches lead to the identification of effects that often fail to replicate [1–3].

In recent years, researchers have increasingly been interested in developing methods to predict the treatment effect for individual patients based on all baseline covariates (features). The importance of individual treatment effects in randomized clinical trials are, for example, argued in Gadbury et al. [4] who proposed identifiable bounds for the proportion of patients in the population that responds favorably to one of the treatments using data from an unmatched 2 by 2 table and discuss the advantages to matching in a matched-pairs design. Dorresteijn et al. [5] predict treatment effects for individual patients and then evaluate the net benefit of making treatment decisions for individual patients based on a predicted absolute treatment effect. Van der Leeuw et al. [6] discusses an individual estimate of the absolute risk reduction in cardiovascular events given the specific combination of clinical characteristics of a patient, while Lamont et al. [7] define Predicted Individual Treatment Effects (PITE) and introduce the PITE framework on which we will base our discussion.

In order to obtain estimates for these individual treatment effects, often traditional statistical models, such as linear regression models are used. While some proposals for using modern machine learning (ML) approaches and artificial intelligence (AI) models, such as regression trees [8], artificial neural networks [9] and Gaussian processes [10], exist (e.g. [11, 12]) their use to date is mostly restricted to illustrative examples and few practical applications exist. In this work, we aim to illustrate the huge potential for ML and AI methods when predicting individual treatment effects and discuss open questions and opportunities for further research.

We will use the PITE framework [7], a simple intuitive framework, to illustrate the potential for ML and AI methods when predicting individual treatment effects. We use PITE as one member of a wide class of different methods that aim to estimate individual treatment effects. It has been chosen to show the huge potential of these methods due to it being simple and intuitive, yet able to encompass many ML methods within, not because it will be the best approach to take in all circumstances. Moreover, PITE has been used in at least two real applications in the past [13, 14]. The highlighted opportunities and challenges, however, exist for other approaches to predicting individual treatment effects as well.

## The PITE Framework

In a clinical trial, we typically observe the outcome for a given patient only under either the experimental or the control condition. Potential outcomes [15–17] provide a powerful framework to overcome this and enable understanding of causal effects – even on an individual level. In a clinical trial, for example, each individual participant has a potential outcome under both the experimental treatment, *Y*_*Ei,*_ and control, *Y*_*Ci*_. The causal effect of an individual can then be defined as *Y*_*Ei*_ – *Y*_*Ci*_. As the outcome is typically only observed under one treatment condition in a clinical trial, however, researchers typically estimate average treatment effects (ATE) defined as

$$ ATE = E\left( {Y_{E} } \right) - E\left( {Y_{C} } \right) $$

where *Y*_*E*_ is the outcome when receiving the experimental treatment and *Y*_*C*_ under the control treatment, possibly accounting for covariates on a population level.

The PITE framework [7, 18] supposes that the outcome of an individual under a given treatment is a function of underlying characteristics so that we can capture some of the potential outcome by predicting this function. Specifically, it supposes that

$$ Y_{{Ti}}  = f_{T} \left( {x_{i} } \right) + \varepsilon _{{Ti}}  $$

where *T* denotes the treatment (E for the experimental group and C for control), *x*_*i*_ is a vector of covariates for individual *i*, ε_Ti_ ~ N(0, σ^2^_T_) is a patient-level random effect and f_T_(.) is an unknown function. Using an estimate of the unknown functions, $\widehat{{f_{T} }}\left( {x_{i} } \right)$, the predicted individual treatment effect (PITE) of a patient *i* is defined as

1 $$ PITE_{i}  = \hat{Y}_{{Ei}}  - \hat{Y}_{{Ci}}  = \hat{f}_{E} \left( {x_{i} } \right) - \hat{f}_{C} \left( {x_{i} } \right) $$

It therefore is an estimate of the individual treatment effect given the covariates and method of estimation of the underlying functions $\widehat{{f_{T} }}\left( . \right)$. Note that, even if the ATE equals zero, there will often be individuals who would be expected to benefit from the treatment while others would be expected to do better under control. As a consequence, PITE can be useful to help guide treatment decisions. Also note that PITEs are estimates of causal effects under conditions typical to those in a randomized controlled trial [19]. Finally it is worth pointing out that for the definition (1) to be valid the variability in the patient-level random effect does not have to be equal to yield unbiased estimates.

## Machine Learning and PITE: An Illustration

To illustrate the PITE framework, we will consider Amyotrophic Lateral Sclerosis (ALS, also known as Motor Neuron Disease), a neurodegenerative disorder that affects motor neurons in the brain and spinal cord and will use the publicly available Pooled Resource Open-Access ALS Clinical Trials database (PRO-ACT, http://nctu.partners.org/ProACT) [20]. People with ALS have progressive weakness in voluntary muscle which affects movement of arms and legs but also impacts speech, swallowing and breathing. The PRO-ACT database includes complete information from close to 3000 patients with ALS who participated in 23 clinical trials of the drug Riluzole. The pooling of multiple randomized trials results in a large enough dataset to obtain predictions even for this rare disease yet due to unaccounted study to study differences, the findings presented here should be viewed as illustrative.

One of the outcome measures often used in ALS is the ALS Functional Rating Scale (ALSFRS) which comprises of a list of 10 different assessments of motor function each of which is scored 0 to 4 (4 = normal function and 0 = no function). The sum of the 10 assessment questions is the ALSFRS score and is measured repeatedly over time for each patient. Following Küffner et al. [20] we use the slope of the ALSFRS score from a repeated measures model for each patient as the outcome of this study. We note that two other studies have used these data to investigate treatment effect heterogeneity [21, 22] using different estimators, both finding evidence for significant individual differences.

In line with [22] we focus on the 2,910 patients (1,766 on experimental treatments and 1,144 on control) who had complete data for 17 predetermined covariates, treatment condition, and the outcome. In this illustration, we will use Bayesian Additive Regression Trees (BART) [23] to estimate the unknown underlying patterns and use 1000 permutations for testing for the existence of heterogeneity. BART has the advantage over the linear model presented in [22] that it can predict well when higher order interactions of non-linearity are present, is robust to outliers in the data, and can handle high-dimensional data without overfitting.

**Fig. 1:**
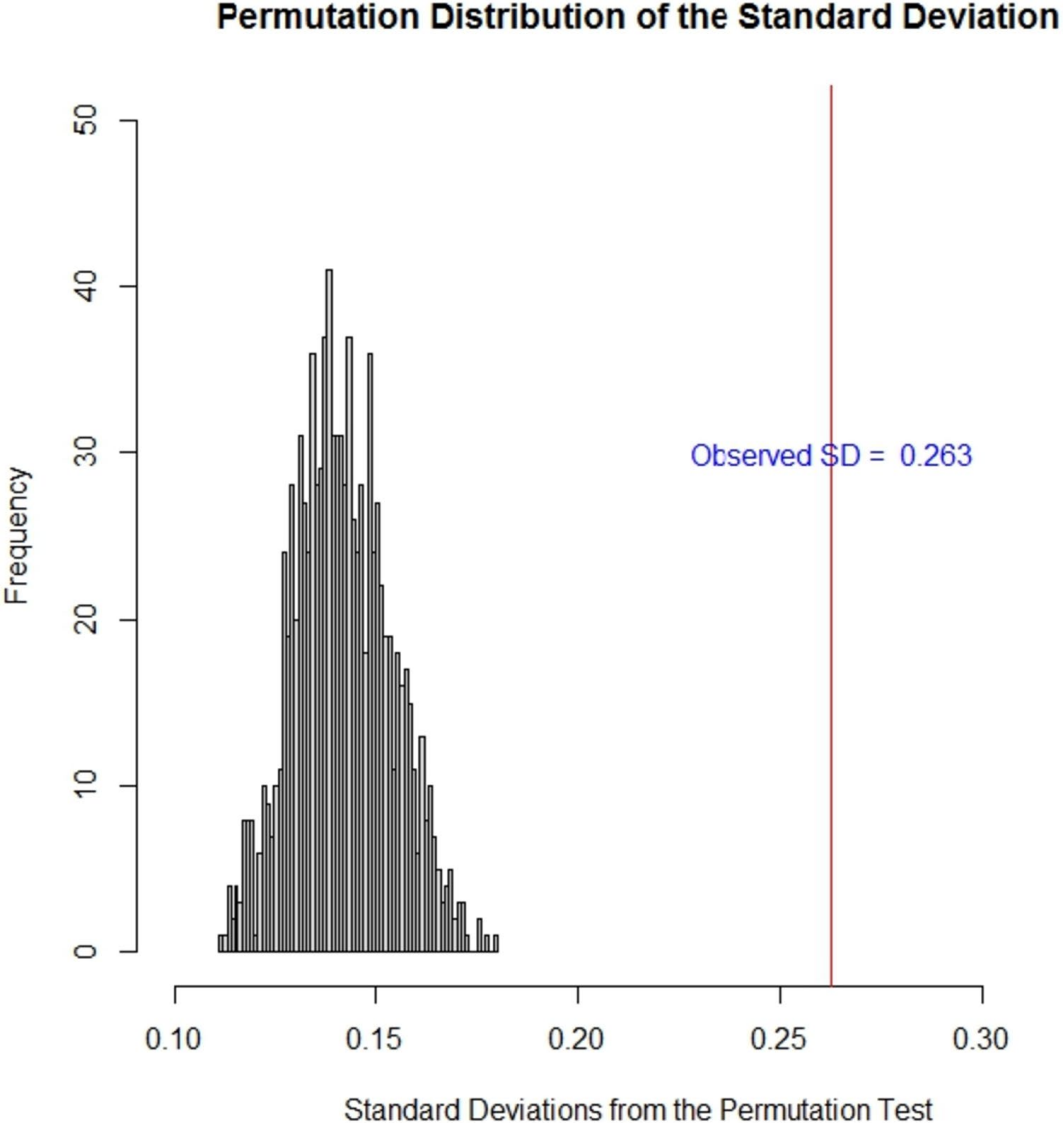
Permutation distribution for the testing for the presence of treatment effect heterogeneity in the ALS dataset using BART as the predictive approach. Vertical red line shows the observed standard deviation resulting in a p-value < 0.001 of the hypothesis of no heterogeneity of effects

*Results* To ensure that a PITE analysis is meaningful, we begin by testing for the presence of treatment effect heterogeneity using a permutation test [22]. Figure 1 confirms that there is strong evidence (p < 0.001) against the hypothesis of no treatment effect heterogeneity in the PITEs using BART as the predictive model. To be consistent with [22], we used the standard deviation of predicted PITE values to define the treatment effect heterogeneity. Interestingly, the standard deviation for the PITEs using BART is greater than those for the linear model reported in [22] suggesting that meaningful higher-order interactions or non-linearity are present. This allows for better prediction, and highlights one of the potential benefits of ML and AI methods in the context of PITE.

**Fig. 2:**
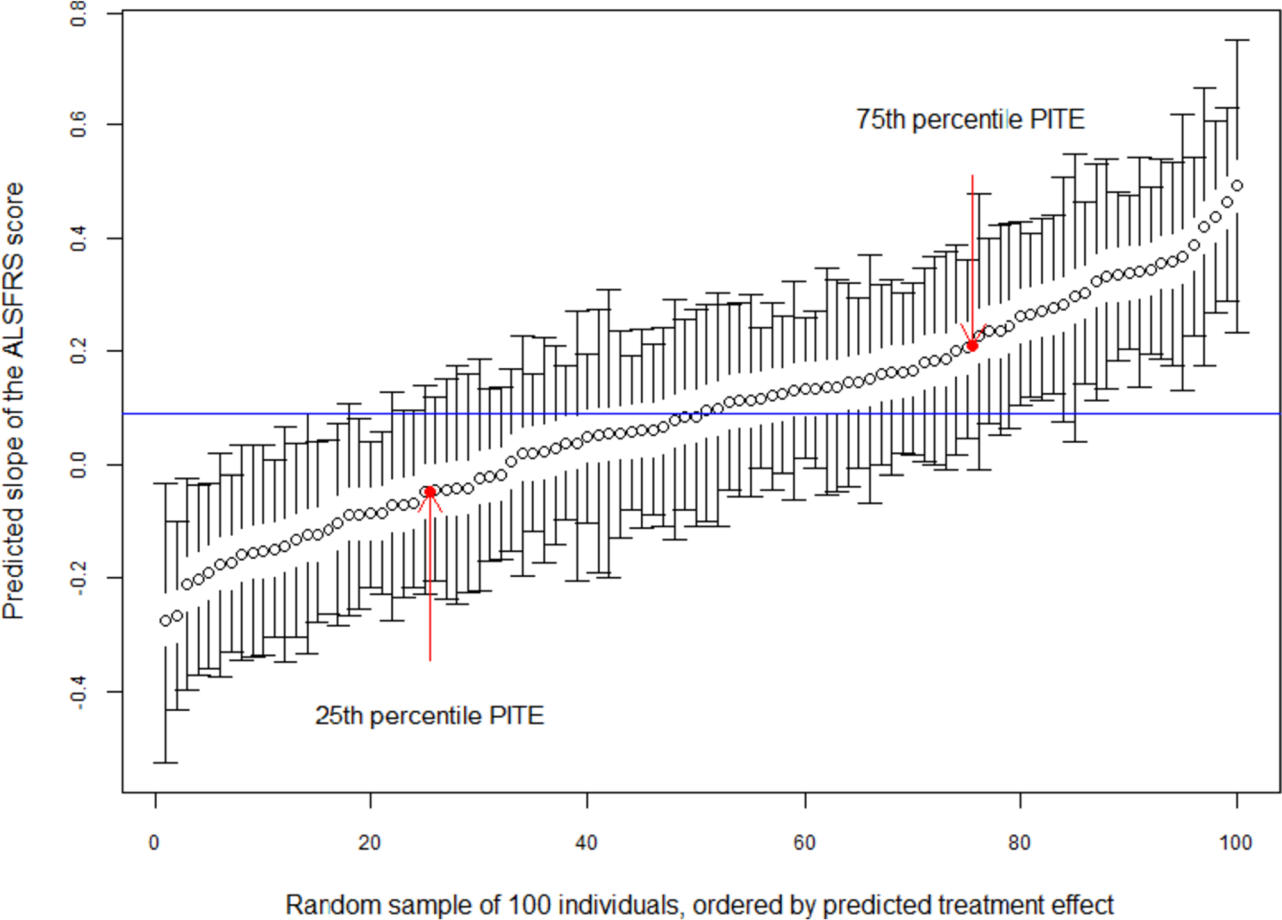
Ordered predicted individual treatment effects together with their 80% intervals for 100 randomly selected patients in the ALS dataset. Horizontal line indicates the average treatment effect

The predicted PITEs are highly variable (Fig. 2) and range from a very clear benefit of the experimental treatment to a clear benefit for control. Most strikingly, one can see that, despite having a small benefit on average, for a number of patients the PITE suggests that using the experimental treatment is actually notably worse than control. Figure 2 also clearly illustrates that, if the PITEs and their uncertainty would be used to make treatment decisions, clear recommendations (i.e. intervals not including zero) would arise for about 40% of patients showing the potential power of such approaches to transform patient care.

A further step in the analyses might be to consider the importance of individual variables. We do not consider this step here as we simply wish to show the potential of these methods to inform treatment decisions rather than provide mechanistic insights for the particular application considered. Moreover we note that it often is to be expected that individual differences are due to accumulating small contributions of many factors rather than big effects of a limited number of factors limiting the utility of variable importance measures.

## Challenges and Opportunities

The illustration above highlights the potential of PITEs to transform treatment of patients by using a patient’s features to inform this patient’s treatment and the flexibility of ML and AI methods make them particularly attractive to use when estimating PITEs. The illustration, however, also gives rise to a number of interesting challenges and under-researched areas, some of which we aim to put in the spotlight below.

*Validation of PITE models* It is widely acknowledged that it is essential for any prediction model to be validated [24, 25] and in an ideal setting, this would occur by evaluating the quality of the predictions in an independent dataset. While initial validation may us alternative approaches, such as sample splitting, independent validation provides much stronger support when PITEs are to be used for decisions in clinical practice.

In the context of personalized predictions based on separate models per treatment condition as in PITE, validation of each separate model, while undoubtedly important, does not automatically imply that the resulting PITEs are valid. To date, limited research has been undertaken to validate individual treatment effect predictions with some very recent suggestions for appropriate metrics being provided in [26].

*Choice of prediction method* From the construction of the PITEs in Eq. (1), it is clear that a necessary condition for them to be identical to the causal effect for an individual is that σ^2^_T_ is zero for both groups and that the estimates $\widehat{{f_{T} }}\left( . \right)$ are equal to the true underlying *f*_*T*_*(.)*. In order to maximise the utility of PITE it is therefore paramount to predict the unknown functions, *f*_*T*_*(.),* as accurately as possible. In general, the PITE framework can utilize any method that allows predicting outcomes on a patient level yet it is to be expected that some methods will perform better than others for a given true underlying data structure. A linear model in the covariates is expected to work very well if there are no non-linear relationships between the covariates and the outcome of interest and all relevant interactions are included in the model. At the same time, such a model will perform poorly in the presence of non-linear effects and higher order interactions which are not included.

In the case of simple linear models, it is well understood when these models are expected to perform well and different diagnostics have been developed to assess the appropriateness of these models. For many ML and AI methods, however, it is less clear if a particular method is suitable, robust and precise for specific underlying relationships and diagnostics to assess this are sometimes lacking. As a consequence, there is a need to develop more and better diagnostics that allow assessing if a particular predictive approach is appropriate.

Moreover, the fact that any predictive approach could be used for the construction of PITEs poses the yet unsolved question of which predictive approach is best for a given application. While one would expect ensemble methods [27] to perform well in general, a rigorous framework for choosing the best approach for a given setting is still missing.

*Utility of PITE to guide treatment* When developing a pharmacological intervention, a high level of evidence is required to show that the intervention is safe and beneficial to patients, and commonly agreed standards apply (e.g., use of two pivotal, well-powered studies in the confirmatory phase), no such standard exists to evaluate the potential added benefit of treatment guided by, for example, PITEs. In principle, randomised clinical trials can be used to evaluate the added benefit for an algorithm as recently done in [28] and some guidance on how to do so exists [29]. Frequently, however, prediction models are updated based on accumulating data so that such (potentially large) evaluations would be required repeatedly—every time a change occurs. Open questions that remain include: (i) when re-evaluation is necessary, and (ii) how to repeatedly assess the benefit of the algorithm if re-evaluation is needed.

*Covariate selection* When trying to best approximate the outcome of a particular patient using ML and AI methods one expects that inclusion of many covariates yields best results. At the same time, one expects that the inclusion of covariates that do not contribute meaningfully to the predictions may add noise to the prediction and, possibly more importantly, incur an unnecessary cost (both monetary and in terms of burden to the patient and/or staff) when collecting the data. Consequently, there remains a desire for the underlying models to be as parsimonious as possible, and a plethora of approaches exist that allow feature selection. In the context of PITE, however, where separate models are used to predict under each treatment condition, there is an opportunity to develop overarching feature selection methods that yield better PITE predictions than when selecting the features separately for each model.

A related area that deserves further attention anchors around the acceptability of these individual treatment predictions by the treating clinician. As one can expect resistance to use such a tool to decide between treatment options when the underlying rationale for the prediction is not well understood, explainable ML and AI methods have a particularly important role to play in this setting. We have also previously argued that utilizing a clinical advisory group to select covariates based on previous research and clinical knowledge can effectively reduce the number of covariates included and improve the acceptability of results [13].

*Responsibility and liability* One final point to raise involves the risk associated with making treatment decisions. Invariably the decision to favor one treatment over another will be incorrect for some patients which poses a question around the responsibility and (potentially) liability. While one can argue that this problem exists with all devices to support treatment decisions, the fact that ML and AI algorithms often are a black box to the medical professional amplifies the issue as the reason for a particular result of the AI approach is not apparent to the user. This question is further amplified when considering situations where the treating clinician deviates from the recommendation made by the algorithm. We believe that more work is urgently needed to clarify the responsibilities and liabilities of individual treatment predictions (and algorithms more generally) in the context of healthcare.

## Discussion

ML and AI methods have a huge potential to transforming healthcare. In this work, we highlight predicting individual treatment effects as one area where ML and AI methods have a potentially large role to play as the flexibility of these methods implies that they are useful with no or limited assumptions about the underlying data structure. In our illustrative example we find that BART, as a representative of ML and AI approaches, showed greater individual differences than simpler linear prediction models, emphasizing the need to consider alternative flexible and robust prediction methods. Before a paradigm shift in which treatment decisions are informed by predictions for individual patients can take place and such modern approaches become widely used, however, and we believe a number of crucial areas deserve further attention. In this work, we have highlighted a few of the most important areas in need for further research and consensus.

## Data Availability

The data are availabe from http://ncri1.partners.org/ProACT/.
